# Evaluation of the Smokeless Tobacco Awareness, Attitude, and Response Knowledge (STAARK) Scale With Integrated Risk Assessment for Tailored Intervention

**DOI:** 10.7759/cureus.70744

**Published:** 2024-10-03

**Authors:** Abigail Rai, Mahesh Kolli, Chao Yuan Li Cai, Samuel Rai, Shyam Nikethen Girivasan

**Affiliations:** 1 General Medicine, Impur Christian Hospital, Mopungchuket, IND; 2 Telemedicine, Apollo Hospitals, Chennai, IND; 3 General Medicine, West Suffolk Hospital, Bury St Edmunds, GBR; 4 Psychiatry, ACS Medical College and Hospital, Chennai, IND; 5 Pharmacy, JSS Academy of Higher Education and Research, Mysuru, IND

**Keywords:** staark scale, smokeless tobacco response, smokeless tobacco attitude, cronbach's alpha in public health, smokeless tobacco awareness, integrated risk assessment (ira), gutka health risks, smokeless tobacco (slt) awareness

## Abstract

Background

Smokeless tobacco (SLT) use, particularly gutkha, is prevalent in India, with cultural acceptance contributing to widespread use, especially among tribal populations. Despite awareness programs, there is limited assessment of their effectiveness, leading to continued vulnerability, even among educated groups. This study aims to evaluate public health strategies by developing and validating the SLT Awareness, Attitude, and Response Knowledge (STAARK) scale, which assesses knowledge and attitudes post-awareness programs on SLT usage through questionnaires, identifying vulnerable individuals, and enabling targeted interventions to reduce SLT use and associated health risks, particularly through integrated risk assessment (IRA).

Methods

This study was performed during an SLT awareness program in Nagaland, utilizing a comprehensive survey to assess participants’ demographics, knowledge, and attitudes. The STAARK score was calculated by combining normalized attitude and knowledge scores, ranging from 0 to 10, categorizing participants’ comprehension and attitudes toward SLT usage awareness. The obtained scores were assessed twice for consistency evaluation of the scale, and vulnerable individuals were subjected to IRA for targeted intervention recommendations. Reliability and correlation analyses were performed, with Cronbach’s alpha validating the scale’s internal consistency.

Results

About 182 participants took up the assessment, with only 180 completing the survey among the population attending the awareness program. Demographic data revealed a diverse participant group, with ages ranging from 13 to 42 years. The survey assessed participants' knowledge and attitudes toward SLT, scoring them on a 0-10 scale for knowledge and a 10-60 scale for attitude. The STAARK scale was developed to combine these scores, providing a comprehensive measure of participants' understanding and attitudes toward SLT. The mean STAARK score was 5.27 with an observed lower limit of 2.40 and an upper limit of 6.50 in the responded population. Reliability testing using Cronbach's alpha showed acceptable internal consistency, though attitude had a weaker correlation with the overall STAARK score compared to knowledge. In a follow-up assessment with 88 participants, the STAARK score averaged 5.10, reinforcing the initial findings. The risk assessment of 17 participants with STAARK scores above six identified varying levels of risk, with most falling into the low- or moderate-risk categories. The study highlights the importance of targeted interventions to address SLT usage, particularly in populations with lenient attitudes toward its consumption.

Conclusions

The STAARK scale can serve as a key tool for evaluating SLT awareness programs by assessing participants’ knowledge and attitudes and identifying high-risk individuals requiring personalized interventions for SLT usage cessation. It standardizes the assessment, enabling comparison across groups and times, and helps identify individuals with lenient attitudes toward SLT for targeted interventions. Its reliability ensures consistency, and by tracking changes in scores before and after programs, it assesses impact and guides future improvements. The STAARK scale is crucial for personalized, evidence-based public health efforts to reduce SLT use. However, eradicating SLT requires ongoing commitment, adaptability, and a focus on long-term outcomes to translate knowledge into lasting change.

## Introduction

Loose leaves, plugs, and twists are examples of smokeless tobacco (SLT) chewed or sandwiched between the cheek and gums or teeth. Nicotine and other tobacco compounds are absorbed through the oral tissues. When using tobacco, users may spit out or swallow the accumulated tobacco fluids. This type of tobacco use has a long history and is widespread in many parts of India. However, there are hazardous components in SLT, primarily including 28 recognized carcinogens, of which nitrosamines constitute the most important. Oral cancers are highly prevalent among users, partly due to the substantial health hazards posed by these toxins. SLT use is influenced by socio-cultural factors, including its role in expressing ethnic identity and low awareness of health risks, with many users believing in its purported health benefits [1,2]. Gutkha is an SLT product from India, made from a mix of tobacco, crushed areca nuts, spices, and other ingredients known to cause nicotine addiction and various cancers [3]. Areca nut and tobacco are both often combined. The majority of individuals are not aware of areca nuts' negative consequences. Areca nut is recognized to have genotoxic and mutagenic effects on bodily tissues, which can result in a variety of neoplastic and paraneoplastic lesions. Areca nut, after nicotine, alcohol, and caffeine, is the fourth most widely used psychoactive drug worldwide [4,5].

The prevalence of SLT use, including gutkha, is notably high in the northeastern states of India, particularly in Assam, Manipur, Tripura, Meghalaya, Mizoram, and the Khasi Jaintia Hills [6]. These areas have been recognized by spatial analysis as important hotspots for tobacco use in women, with prevalence rising with age, particularly among women 40-49 years old, who have a threefold greater likelihood to use SLT than younger women. Studies highlight that divorced and widowed women, those with low education levels, and women from lower socioeconomic groups are more prone to SLT use. Additionally, religious affiliations and urban residency are associated with higher rates of SLT use. The cultural acceptance of SLT in these regions, combined with low awareness of its health risks, reinforces its widespread use [6-8].

The majority of tobacco users begin using tobacco in their teens and continue throughout adulthood. The World Health Organization states that teenagers are more prone to take risks, which might result in substance misuse. They are still undergoing vital stages of growth and development, which leaves them susceptible to the negative effects of nicotine [9,10]. As per estimates, a lack of cessation programs might result in an extra 160 million smoking-related mortality worldwide by the year 2050. About 70% of tobacco users say they want to give up the habit, but only 3% to 5% of them can do so [11].

In India, SLT consumption is twice as prevalent as smoking, particularly among tribal populations, where it is culturally ingrained. SLT use starts early and is passed down through generations. Among tribal groups, SLT use prevalence is notably high at 63.4%, reflecting its deep-rooted sociocultural acceptance [12]. The National Tobacco Control Program provides support for the expanded implementation and monitoring of the Cigarettes and Other Tobacco Products Act of 2003 [13], which prohibits the purchasing process of tobacco products to minors and near schools, enforces smoking restrictions, and requires health warnings [14].

Awareness programs on SLT usage are widespread in India, while attitude and knowledge assessments post-event are highly limited. This questions the effectiveness of the program, resulting in the vulnerability of the educated population toward SLT consumption. This analysis aims to assess public health awareness strategies and educational campaigns. By understanding the gaps in knowledge and attitude based on educational background, targeted interventions can be designed to reduce gutkha usage and its associated health risks. This study focuses on generating and validating the SLT Awareness, Attitude, and Response Knowledge (STAARK) scale, which evaluates the knowledge and attitude of the population who attended the awareness program using a questionnaire-based survey at different points in time. This study further analyzes vulnerable populations toward their tobacco consumption risk using integrated risk assessment (IRA) and the need for personalized care.

## Materials and methods

This cross-sectional study was conducted during an SLT usage awareness program held at a secondary care hospital in Nagaland. Ethical clearance was obtained from the Institutional Review Board of Impur Christian Hospital (approval number: 2024/0001/IRB-ICH) toward the conduct of the study, recognizing it as a minimal-risk research project, using a questionnaire-based survey focused on assessing knowledge and attitudes toward SLT usage within the population. Participants were asked to complete a comprehensive survey designed to assess their demographics, knowledge, and attitudes toward SLT. The study population consisted of participants from the awareness program, and the inclusion criteria required individuals aged between 13 and 60 who consented to participate in the survey, while those who declined or failed to complete the survey were excluded. A target sample size of 182 participants was set based on the prevalence rates of SLT use and calculations designed to ensure adequate statistical power for correlation analyses between knowledge and attitudes. This survey was administered at two different times following the awareness program to gauge changes over time and assess the reliability of the scale. The responses for the survey were collected using Google Forms (Mountain View, CA, USA), an online web-based platform chosen for its ease of access by the participants with smartphones.

Survey scoring assessment

The survey consisted of three main sections (Appendix 1). The first section gathered demographic information, including age, height, weight, and highest educational qualification, with a total of four questions. This data provided a detailed profile of the participants. The second section evaluated the participants' attitudes using 10 questions rated on a Likert scale. Three questions utilized a five-point scale, while the remaining seven used a four-point scale. The total attitude score, ranging from 10 to 60, was classified into five categories: 10-19 for a strong avoidance or highly negative attitude, 20-29 for a moderate avoidance or negative attitude, 30-39 for a neutral attitude, 40-49 for a moderately lenient attitude, and 50-60 for a strongly lenient or positive attitude for SLT usage. Higher scores demonstrated a more lenient attitude toward gutkha usage. These scores were further scaled for attitude and credited with one point for a strong avoidance/highly negative attitude, two for a moderately negative attitude, three for a neutral attitude, four for a lenient attitude, and five for a strong positive attitude toward SLT usage. The third section assessed knowledge with 10 multiple-choice questions. Each question had four possible answers, with only one correct option. Knowledge scores ranged from 0 to 10, categorized as follows: zero to three indicating low knowledge, four to six representing moderate knowledge, seven to eight reflecting good knowledge, and nine to 10 denoting excellent knowledge. These categories were then scaled for knowledge on a point basis as one for low knowledge, two for moderate knowledge, three for good knowledge, and four for excellent knowledge, which helped in understanding the depth of the participants' comprehension regarding gutkha, including its ingredients, risks, prevention strategies, and rehabilitation methods.

The simplification was implemented to make the STAARK scale more accessible for large-scale public health interventions where detailed scoring might be impractical. It allows for quick interpretation and helps identify patterns in knowledge and attitude more easily. The 1-5 scale still captures key differences in knowledge and attitude but presents them in a format that is easy for practitioners and policymakers to interpret. This system retains the original intent of assessing both awareness and leniency toward SLT use while reducing complexity for practical application in targeted interventions. While the original 10-60 scale provides detailed insights into participants' knowledge and attitudes, the 1-5 scale offers a streamlined version that retains essential information while making the results more actionable for public health initiatives.

To analyze the data, descriptive statistics were first applied to the demographic information to provide a summary of participant characteristics. The knowledge and attitude scores were then correlated to determine the relationship between participants' understanding and their attitudes. Attitude scores were normalized using the formula

normalized attitude score = [(attitude score-10)/50] X 10

This normalization allowed for a standardized comparison with knowledge scores. The final STAARK score was calculated by combining the normalized attitude score with the knowledge score.

STAARK score

The STAARK score, ranging from 0 to 10, was interpreted as follows: 0 to two indicated a poor understanding and negative attitude toward gutkha issues, 2.1 to four reflected fair knowledge and avoidance attitude toward SLT, 4.1 to six denoted good knowledge and a generally neutral attitude toward SLT, 6.1 to eight indicated very good understanding and a lenient attitude toward SLT usage, and 8.1-10.0 represented excellent grasp and a strongly positive stance toward addressing SLT usage.

STAARK = (normalized attitude score + knowledge score)/2

IRA

The IRA consisted of seven questionnaires with varying point system grading from 0 to 41 (Appendix II). Scoring was performed on a set of seven questionnaires where the participants were categorized based on scores as low risk having scores between 0 and 10 points, moderate risk having scores between 11 and 20 points, high risk having scores between 21 and 30 points, and very high risk having scores between 31 and 41 points. Only the population in Assessment I (Results section) having a STAARK score above 6.1 was subjected to risk assessment due to the presence of a lenient attitude toward gutkha usage.

Statistical analysis

The statistical analyses were performed using SAS Studio version 9.4 (SAS Institute Inc., Cary, NC, United States), which facilitated the execution of descriptive statistics, correlation analysis, and reliability testing. Pearson correlation was used for correlating the data. A reliability analysis was conducted to validate the final STAARK score. Cronbach’s alpha was used to measure the internal consistency of the scale, ensuring that the items within the scale reliably assessed participants’ knowledge and attitudes toward gutkha.

## Results

Sample demographics

A total of 182 participants responded to the awareness program, of which two respondents did not complete the survey. Table 1 shows the descriptive statistics on the demographics of the participating population. The participants had a mean age of 23.82 years, a standard deviation of 6.98 years, and a broad age range from 13 to 42 years, indicating variability in the age distribution. Height measurements show an average of 157.97 cm with a standard deviation of 8.59 cm and scores ranging from 125.27 cm to 180 cm. This wide range suggests significant diversity in stature among the participants. The mean weight for the recorded respondents was found to be 51.30 kg, with a standard deviation of 9.74 kg, and weights spanning from as low as 25.30 kg to as high as 98 kg. The range in weight, along with the standard deviation, further underscores the heterogeneity of the sample.

**Table 1:** Descriptive statistics on the participants N: total number of individuals, Std. Dev.: standard deviation, cm: centimeters, Kg: kilograms

Demographic	N	Mean	Std. Dev.	Minimum	Maximum
Age (years)	180	23.82	6.98	13.00	42.00
Height (cm)	180	157.97	8.59	125.27	180.00
Weight (Kg)	180	51.30	9.74	25.30	98.00

**Figure 1:**
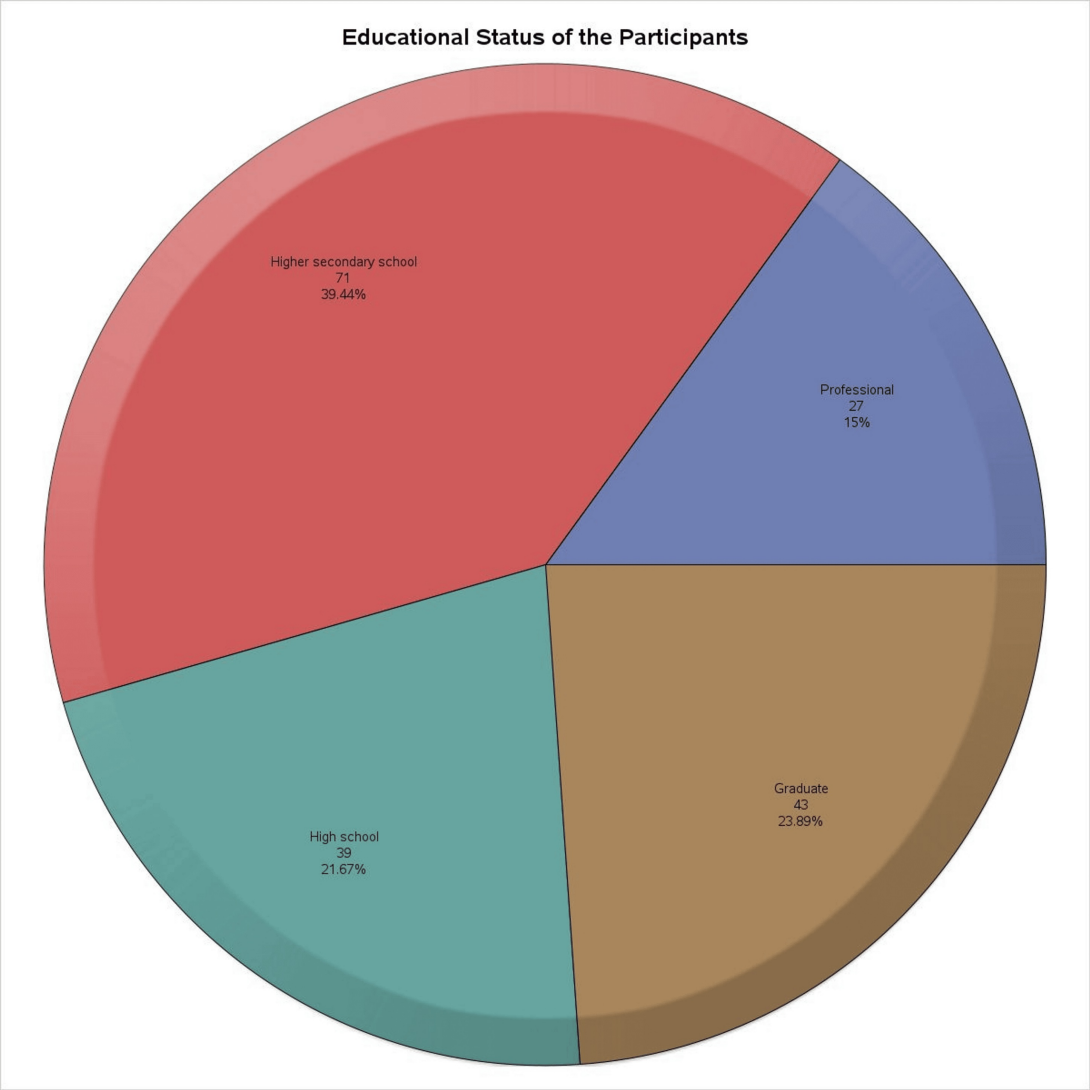
Highest educational qualification of the study participants The pie chart illustrates the educational status distribution of the participants in the study. The segments represent different educational levels: higher secondary school (71 participants), high school (39 participants), graduate (43 participants), and professional (27 participants). Each segment is color-coded to distinguish between the categories, with the largest portion of participants being from the higher secondary school level. This distribution helps in understanding the educational background of the study's respondents.

The pie chart in Figure 1 illustrates the educational status of the participants. The four segments represent different educational levels. The largest segment comprises participants with a “higher secondary school” education, totaling 71 individuals. The second-largest group consists of “graduates” with 43 participants. “High school” participants account for 39 individuals, while the smallest group, labeled “professional,” includes 27 participants. The chart effectively conveys the distribution of educational backgrounds within the sample population.

Assessment I

Knowledge Scoring

**Figure 2:**
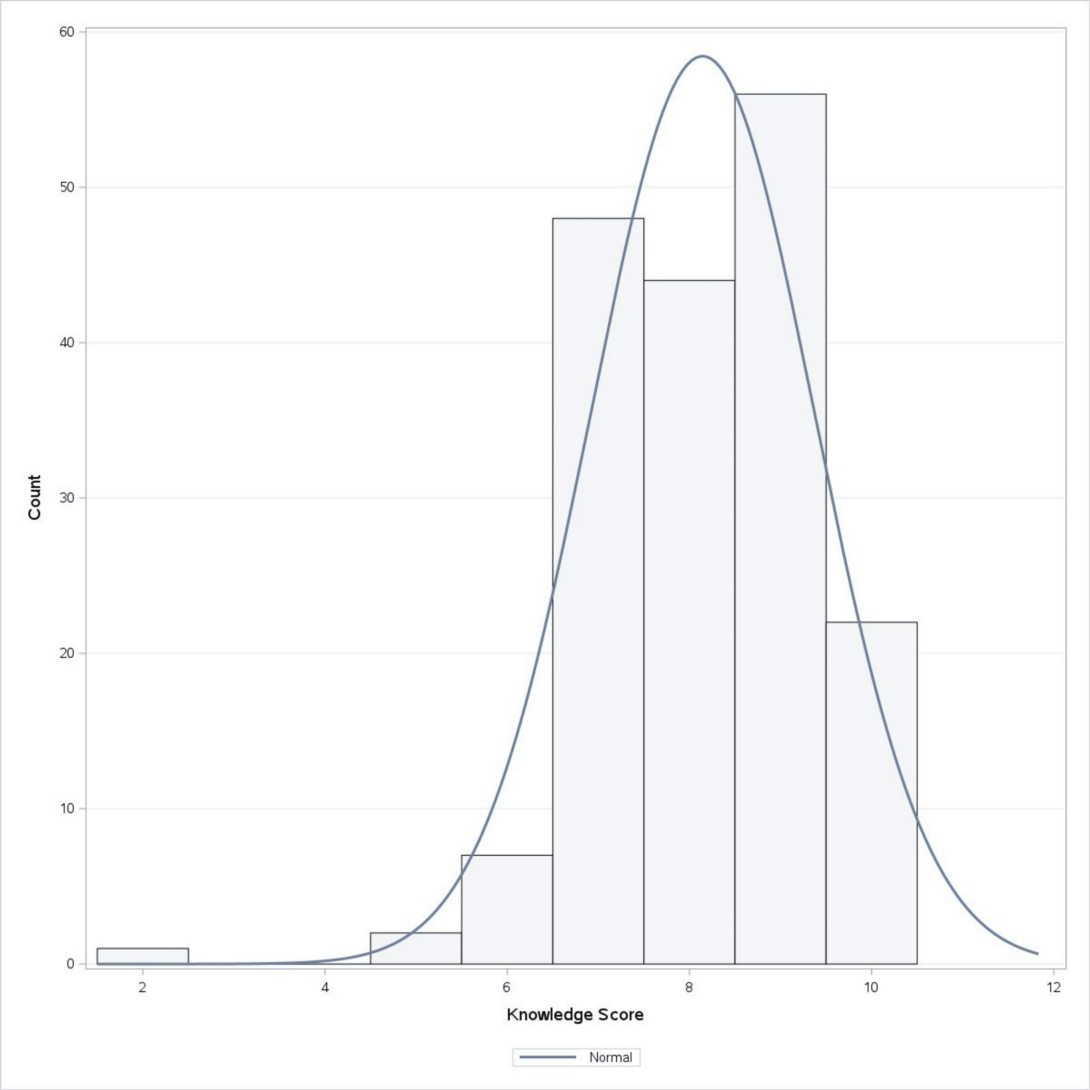
Frequency of knowledge scoring for Assessment I The histogram displays the distribution of the knowledge score among participants, with the x-axis representing the knowledge score and the y-axis representing the count of participants. The superimposed line represents the normal distribution curve for comparison, showing how closely the data approximates a normal distribution.

The participants’ responses were calculated for knowledge scoring, which provided a score ranging from 0 to 10 points. Figure 2 describes the frequency of the scores obtained from the 180 participants. Thirty-three percent of the participants obtained a score of nine, indicating the highest frequency. Overall, the average knowledge score of the participants was 8.14 with a standard deviation of 1.23.

The knowledge score was further scaled on a four-point system, which is represented as a knowledge scale, as described in Table 2. A score of three was the highest scaled score among the population, indicating good knowledge of the awareness program contents.

**Table 2:** Knowledge scale frequency for Assessment I Knowledge scores were scaled on a point basis as one for low knowledge, two for moderate knowledge, three for good knowledge, and four for excellent knowledge, which helped in understanding the depth of participants’ comprehension.

Knowledge scale	Frequency	Percentage (%)	Cumulative frequency
1	1	0.56	3
2	9	5.00	12
3	92	51.11	104
4	78	43.33	180

Attitude Scoring

**Figure 3:**
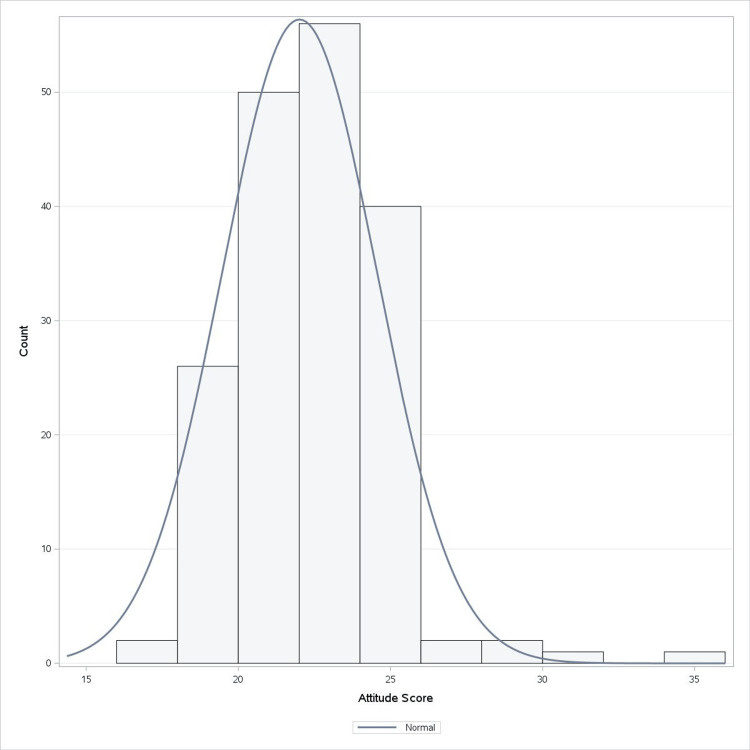
Frequency of attitude scoring for Assessment I The histogram illustrates the distribution of the attitude total score among participants, with the x-axis representing the attitude total score and the y-axis showing the count of participants. The blue line represents the normal distribution curve, allowing for a visual comparison of how the data aligns with a normal distribution. This visualization helps assess the overall attitude of participants toward the subject matter, as indicated by their scores.

The attitude questionnaire included a two-factor assessment: six questions assessing the attitude toward health and social impacts and four questions assessing the attitude toward public policy and education. The attitude scores were calculated from the responses, which were on a Likert scoring basis with a minimum score of 10 and a maximum score of 60. Figure 3 represents the attitude score frequency ranging from 16 to 35 for the participants included in our study with a mean score of 22 with a standard deviation of 2.55.

The scores obtained were further subjected to attitude scaling under a five-point scale, which yielded results as described in Table 3. A score of two was the highest scaled attitude obtained, indicating a moderately negative or avoidance attitude toward SLT usage.

**Table 3:** Attitude scale frequency for Assessment I Attitude scores were further scaled and credited with one point for a strong avoidance/highly negative attitude, two for a moderately negative attitude, three for a neutral attitude toward SLT usage, four for a lenient attitude, and five for a strong positive attitude toward SLT usage. SLT: smokeless tobacco

Attitude scale	Frequency	Percentage (%)	Cumulative frequency
1	28	15.56	28
2	150	83.33	178
3	2	1.11	180
4	0	0.00	180
5	0	0.00	180

STAARK Scoring

**Figure 4:**
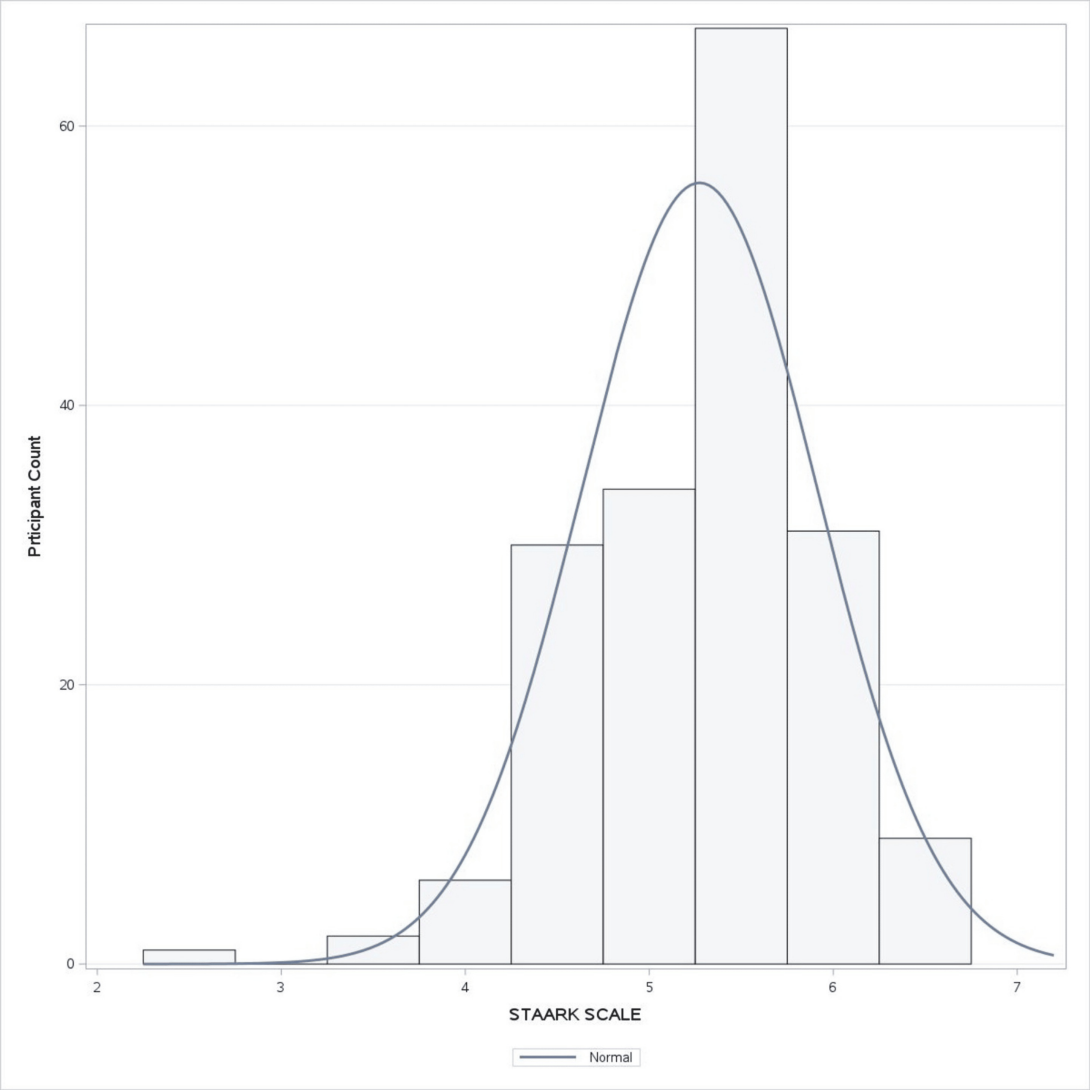
STAARK scale frequency The histogram illustrates the distribution of the STAARK scores among participants. The x-axis represents the STAARK scores, and the y-axis shows the number of participants. The blue line overlay represents the normal distribution curve, providing a visual comparison of the data's alignment with a normal distribution and highlighting the overall spread and central tendency of the participants' scores. The STAARK score, ranging from 0 to 10, was interpreted as follows: 0 to two indicated a poor understanding and negative attitude toward gutkha issues, 2.1 to four reflected fair knowledge and avoidance attitude toward SLT, 4.1 to six denoted good knowledge and a generally neutral attitude toward SLT, 6.1 to eight indicated very good understanding and lenient attitude toward SLT usage, and 8.1-10.0 represented excellent grasp and a strong, positive stance toward addressing SLT usage. STAARK: smokeless tobacco awareness, attitude, and response knowledge, SLT: smokeless tobacco

The STAARK scale was performed from the knowledge and attitude scores by normalizing the attitude score and calculated as per the formula mentioned in the Materials and Methods section. Figure 4 describes the overall frequency of the STAARK scores on the participants ranging from 0 to 10, with a mean score of 5.27 and a standard deviation of 0.64.

STAARK Score Validation

The descriptive statistics for the variable’s knowledge scale, attitude scale, and STAARK scale provide a clear overview of the data distribution across 180 observations as described in Table 4. The mean score for the knowledge scale was found to be 3.37 with a standard deviation of 0.61, indicating relatively close clustering of scores around the mean. The scores range from one to four. For the attitude scale, the mean score was found to be 1.86 with a standard deviation of 0.38, showing a smaller spread, with scores ranging from one to three. The STAARK scale has a mean score of 5.27 and a standard deviation of 0.64, with a range between 2.40 and 6.50.

**Table 4:** Descriptive statistics on reliability assessment parameters for Assessment I N: number of participants, Std. Dev.: standard deviation, STAARK: smokeless tobacco awareness, attitude, and response knowledge

Variable	N	Mean	Std. Dev.	Sum	Minimum	Maximum
Knowledge scale	180	3.37	0.61	607.00	1.000	4.00
Attitude scale	180	1.86	0.38	334.00	1.00	3.00
STAARK	180	5.27	0.64	949.20	2.40	6.50

The reliability assessment using Cronbach's alpha indicates acceptable internal consistency among the variables, which is described for Assessment I in Table 5 and Table 6 (for deleted variables). The raw Cronbach's alpha was found to be 0.70 (0.698, which approximates 0.70), and the standardized alpha was slightly lower at 0.66, suggesting that the items collectively measure a common construct with moderate reliability. The STAARK scale shows a strong correlation with the total (0.86), indicating it is a key contributor to the overall consistency of the scale.

**Table 5:** Cronbach alpha for Assessment I A Cronbach’s alpha of less than 0.40 is considered unacceptable. A Cronbach's alpha of 0.7 or above suggests that the questionnaire has strong reliability. The raw value is based on item co-variance, and the standardized value is based on item correlation.

Variables	Alpha
Raw	0.698563
Standardized	0.655550

**Table 6:** Cronbach's alpha with deleted variable for Assessment I The raw value is based on item co-variance, and the standardized value is based on item correlation. STAARK: smokeless tobacco awareness, attitude, and response knowledge

Deleted variable	Raw variables		Standardized variables	
	Correlation with total	Alpha	Correlation with total	Alpha
Knowledge scale	0.628368	0.447378	0.513686	0.493363
Attitude scale	0.184614	0.900782	0.180132	0.901532
STAARK	0.862605	0.028946	0.805356	0.032034

Table 7 describes the Pearson correlation coefficients of the variables. The Pearson correlation coefficients reveal strong positive correlations, particularly between the knowledge scale and the STAARK scale (r=0.82072, p<0.0001), indicating a significant relationship. The correlation between the attitude scale and the other variables is weaker but still significant, highlighting different levels of association across the variables. There is a strong positive correlation between knowledge and STAARK scores, indicating that higher knowledge about gutkha corresponds with increased STAARK scores. Similarly, a moderately positive correlation between attitude and STAARK scores in Assessment I suggests participants with more lenient attitudes toward gutkha also have higher STAARK scores.

**Table 7:** Pearson correlation coefficients for Assessment I Probability > |r| under H0: Rho=0 The p-values are indicated in the parenthesis '()', which describes the correlation between the variables. There is a strong and significant positive correlation between knowledge scores and STAARK scores, which means that as participants' knowledge about gutkha increases, their STAARK score (a combined measure of knowledge and attitude) also increases. There is a moderate and significant positive correlation between attitude scores and STAARK scores. This indicates that participants with more lenient or positive attitudes toward gutkha tend to have higher STAARK scores. STAARK: smokeless tobacco awareness, attitude, and response knowledge

	Knowledge scale	Attitude scale	STAARK scale
Knowledge scale	1.00000	0.01628 (0.8283)	0.82072 (<0.0001)
Attitude scale	0.01628 (0.8283)	1.00000	0.32746 (<0.0001)
STAARK	0.82072 (<0.0001)	0.32746 (<0.0001)	1.00000

Risk Scores

The observed results from 180 participants were further filtered based on the STAARK scale, yielding a list of lenient and positive attitudes (STAARK >6.1) in the population toward SLT consumption and were subjected to risk assessment. Seventeen participants with a STAARK score higher than 6.1 were assessed for risk scores as described in Table 8. Four individuals in the high-risk category scored between 21 and 30, six individuals in the moderate-risk category scored between 11 and 20, and seven individuals in the low-risk category scored between 0 and 10.

**Table 8:** Risk score assessment on the STAARK score above 6.1 Low risk having IRA scores (as represented in Appendix 2) between 0 and 10 points, moderate risk having IRA scores between 11 to 20 points, high risk having IRA scores between 21 to 30 points, and very high risk having IRA scores between 31 to 41 points STAARK: smokeless tobacco awareness, attitude, and response knowledge, IRA: integrated risk assessment

Risk category	Frequency	Percent
High	4	23.53
Moderate	6	35.29
Low	7	41.18

Assessment II

Assessment II was performed on 88 respondents of the same population, post two weeks of Assessment I. In the second assessment, the descriptive statistics for the variables “knowledge scale,” “attitude scale,” and “STAARK” were evaluated across 88 participants. The mean knowledge scale was 3.44, with a standard deviation of 0.56, indicating that the participants generally had moderate knowledge about SLT. The attitude scale had a mean of 1.44 and a standard deviation of 0.50, reflecting a predominantly negative attitude toward SLT usage. The STAARK score, which combines both knowledge and attitude scores, had a mean of 5.11 with a standard deviation of 0.52, suggesting a moderate overall understanding and attitude toward SLT. In Assessment II, the hypothesis was that the participants’ STAARK scores would either remain stable or decrease, reflecting improved knowledge and more negative attitudes toward SLT usage after the awareness program. A decrease in the STAARK score would indicate a shift toward greater awareness and less leniency in attitudes, aligning with the program's goals and with reduced memory of knowledge. The observed decrease in the STAARK score, from 5.27 in Assessment I to 5.11 in Assessment II, supports this hypothesis, suggesting that participants developed more cautious attitudes toward SLT use over time. The decrease in the STAARK score must be interpreted in light of its potential to evaluate the reliability of the scale. The consistency of the STAARK score across the two assessments, despite the small variation, indicates the scale's ability to track changes in both knowledge and attitude. Therefore, this decrease was expected and supports the hypothesis that the STAARK scale effectively measures shifts in understanding and attitudes following intervention. Table 9 describes the descriptive statistics on Assessment II parameters.

**Table 9:** Descriptive statistics on reliability assessment parameters for Assessment II N: number of participants, Std. Dev.: standard deviation, STAARK: smokeless tobacco awareness, attitude, and response knowledge

Variable	N	Mean	Std. Dev.	Sum	Minimum	Maximum
Knowledge scale	88	3.44	0.56	303.00	2.00	4.00
Attitude scale	88	1.44	0.50	127.00	1.00	2.00
STAARK	88	5.11	0.52	449.20	3.90	6.20

Table 10 and Table 11 (for deleted variables) outline the reliability and validation of the scale. The reliability of the scales was assessed using Cronbach's alpha. The raw Cronbach's alpha was 0.56, indicating moderate internal consistency among the variables. The low correlation between attitude and the STAARK scale suggests that attitude has a weaker influence on the overall assessment as compared to knowledge, which showed a strong correlation (0.75) with the STAARK score.

**Table 10:** Cronbach's alpha for Assessment II A Cronbach’s alpha of less than 0.40 is considered unacceptable. A Cronbach's alpha of 0.7 or above suggests that the questionnaire has strong reliability. The raw value is based on item co-variance, and the standardized value is based on item correlation.

Variables	Alpha
Raw	0.560583
Standardized	0.552150

**Table 11:** Cronbach's alpha with deleted variable for Assessment II Raw value is based on item co-variance, and standardized value is based on item correlation. STAARK: smokeless tobacco awareness, attitude, and response knowledge

Deleted variable	Raw variables		Standardized variables	
	Correlation with total	Alpha	Correlation with total	Alpha
Knowledge scale	0.402816	0.407187	0.389857	0.407539
Attitude scale	0.057360	0.856888	0.065209	0.858278
STAARK	0.786644	-0.306469	0.765605	-0.309104

Table 12 presents a correlation matrix between the knowledge scale, attitude scale, and STAARK scale, with corresponding p-values for Assessment II. The correlations indicate the strength and direction of the relationships between participants' knowledge and attitudes toward gutkha usage and the overall STAARK score.

**Table 12:** Pearson correlation coefficients for Assessment II Probability > |r| under H0: Rho=0 The p-values are indicated in the parentheses '()', which describes the correlation between the variables. There is a strong and significant positive correlation between the knowledge scale and the STAARK score. This means that participants with higher knowledge about gutkha tend to have higher overall STAARK scores, indicating that knowledge is a key contributor to the STAARK score. There is a moderate and significant positive correlation between attitude scores and STAARK scores, suggesting that as the participant's attitude becomes more lenient toward gutkha usage, their STAARK score increases, although this effect is not as strong as the influence of knowledge. STAARK: smokeless tobacco awareness, attitude, and response knowledge

	Knowledge scale	Attitude scale	STAARK scale
Knowledge scale	1.00000	-0.13386 (0.2137)	0.75174 (<0.0001)
Attitude scale	-0.13386 (0.2137)	1.00000	0.25592 (0.0161)
STAARK	0.75174 (<0.0001)	0.25592 (0.0161)	1.00000

## Discussion

Numerous scales have been developed to measure nicotine dependence, particularly in the context of smoking and SLT use. Among these, 16 scales specifically assess dependence on SLT, with 13 scales originating from the USA, two from Taiwan, and one from Sweden. Notably, the Betel Quid Dependence Scale and Betel Quid Dependence Instrument cater to specific products, highlighting the diversity in approaches to measuring nicotine dependence [15]. While scales like the Fagerstrom Nicotine Dependence Scale for SLT [16] effectively measure dependence, there is a glaring shortage of scales designed to evaluate the effectiveness of SLT prevention programs. The few existing SLT prevention programs often integrate into broader tobacco or substance use prevention efforts, complicating the evaluation of their specific impact on SLT use. Despite initiatives by entities like the Nation Institute of Health to develop youth-focused interventions, especially in school settings, the field still lacks dedicated tools to assess the success of these prevention strategies, underscoring a critical need for more targeted evaluation frameworks [17]. The STAARK scale provides a comprehensive evaluation based on a 10-point system, evaluates the attitude and knowledge of an individual toward such awareness programs, and enables the host to identify the vulnerable population who are on the verge of tobacco addiction. These identified individuals can be further subjected to risk assessment to quantify the tailored counseling or therapeutic management.

Scale construct and reliability

This scale is useful in awareness programs to evaluate the impact of such programs on participants' knowledge and attitudes toward SLT, ensuring that the programs are effectively addressing the issues associated with SLT use. The findings reveal a complex relationship between knowledge, attitude, and the STAARK scale, which effectively identifies educated individuals who continue to use SLT despite their knowledge of its risks. Our analysis shows that the knowledge score is directly proportional to the STAARK score, indicating that individuals with higher levels of awareness about SLT risks are still part of the educated group exhibiting SLT use. This highlights a critical gap between knowledge and behavioral change, where awareness does not necessarily deter SLT consumption. On the other hand, the attitude score is inversely proportional to the STAARK score, demonstrating that individuals with a more cautious or negative attitude toward SLT tend to exhibit lower STAARK scores, reflecting a reduced tendency to engage in SLT use. This inverse relationship suggests that interventions aimed at modifying attitudes might be effective in reducing SLT consumption. Given the prevalence of SLT use among educated populations in urban localities, targeted awareness programs should be designed not only to enhance knowledge but also to shift attitudes more effectively. Such programs would serve as valuable tools for identifying individuals who may benefit from specific intervention strategies aimed at reducing SLT use, particularly in high-risk, educated demographics. This targeted approach could lead to more successful outcomes in SLT prevention and cessation efforts.

To evaluate the participant characteristics, only Assessment I was considered due to the high population count. A STAARK score on this scale would ideally center around a score of five, reflecting a balanced understanding of attitude toward gutkha usage. A score near five signifies a participant who possesses a moderate level of knowledge about gutkha, including its ingredients, risks, and prevention strategies, coupled with a neutral attitude toward its usage. This balanced score is crucial as it indicates a reasonable comprehension of the subject matter without strong bias toward either lenient or avoidance attitudes. Scores above five suggest a lenient attitude, indicating a deeper understanding but possibly a more permissive stance on gutkha use, which could be concerning in public health contexts. Conversely, scores below five reflect a lower level of knowledge but a more cautious or negative attitude toward SLT usage, which, while protective, may lack the informed foundation necessary for effective decision-making or advocacy. Therefore, a score around five is ideal, as it demonstrates a well-rounded and informed stance, essential for individuals who may influence public health outcomes related to gutkha usage. When examining the impact of each variable on the overall reliability, the attitude scale stands out with a low correlation and a significant increase in alpha to 0.90 when it is deleted. This suggests that the attitude scale may not align well with the other variables and could be contributing to noise rather than consistency.

Assessment I, with a larger participant pool, provides a broader and more robust evaluation, offering a more comprehensive view of the knowledge and attitudes toward SLT. The data from Assessment I demonstrate a wider distribution of scores and a more detailed correlation analysis, making it superior in terms of generalizability and depth of insight. The reliability of the STAARK scale was established across a larger dataset, which strengthens the validity of the findings.

However, despite the reduced number of participants in Assessment II, the STAARK scale still exhibited strong internal consistency and significant correlations, particularly between knowledge and the STAARK scale. This consistency across different sample sizes underscores the reliability of the STAARK scale. The scale remains a dependable tool for assessing knowledge and attitudes in SLT-related studies, proving its applicability across various research settings, regardless of participant size. Therefore, the STAARK scale is reliable and effective for any study aimed at evaluating knowledge and attitudes toward SLT usage. The frequency distribution showed that most participants had knowledge scores of 8-9, while attitude scores were clustered around one and two, indicating moderate to strong negative attitudes toward SLT. The STAARK scores mostly ranged between 4.9 and 5.7, indicating generally good but not excellent understanding and attitude toward SLT issues.

A Cronbach’s alpha of less than 0.40 is considered unacceptable, demonstrating that the scale items were inconsistent in measuring the intended constructs. According to several suggestions, a Cronbach's alpha of 0.7 or above suggests that the questionnaire has strong reliability [18]. Dependability is considered satisfactory when it is between 0.4 and 0.75. Good dependability is indicated by a score greater than 0.75 [19,20]. The scale validity is assessed through reliability and correlation analyses in both assessments. For Assessment I, Cronbach's alpha indicates moderate reliability (0.70 for raw, 0.66 standardized), with the STAARK score showing strong internal consistency (0.86) and significant correlation with the overall scale. The Pearson correlations suggest that knowledge has a strong relationship with the STAARK scale (r=0.82, p<0.0001), while attitude's correlation is weaker but still significant. Assessment II displays lower reliability (0.56 raw), and the attitude scale shows minimal impact on the STAARK scale. The Pearson correlation coefficients reinforce that knowledge remains the primary contributor to the STAARK scale (r=0.75, p<0.0001), while attitude's influence is considerably weaker. This suggests that while the scales measure related constructs, knowledge is a more critical determinant of the overall assessment than attitude.

Participant assessments

The analysis of the STAARK scale across different age groups in the study provides insight into the understanding and attitudes toward gutkha usage among teenagers, young adults, and adults. The mean scores for these groups, five for teenagers less than 18 years of age, 5.30 for young adults between 19 and 25 years of age, and 5.45 for adults greater than 25 years of age, indicate varying levels of knowledge and attitudes toward SLT. Teenagers, with a mean score of five, demonstrated a balanced understanding and a neutral attitude toward gutkha usage. This score is close to the ideal score of five, reflecting an awareness of the risks and ingredients of gutkha without significant bias. Young adults, with a slightly higher mean of 5.30, may have a more permissive stance on gutkha use, suggesting a deeper understanding but possibly concerning leniency toward SLT. Adults with a mean score of 5.45 show the highest level of understanding, yet their attitude might be even more permissive, which could raise public health concerns. The higher scores in older age groups suggest that individuals might develop a more informed but potentially lenient attitude toward SLT. This trend underscores the need for targeted interventions, especially among young adults and adults, to promote cautious and informed decision-making regarding gutkha use.

The analysis of the STAARK score based on educational background reveals distinct trends that shed light on how education influences understanding and attitudes toward gutkha usage. The data shows that high school participants have a mean STAARK score of 4.82, indicating a fair level of knowledge and a generally neutral attitude toward SLT. This score suggests that while these participants are somewhat informed, they may lack a comprehensive understanding of the risks associated with gutkha. Higher secondary school participants have a slightly higher mean STAARK score of 5.35. This score reflects a good level of knowledge and a neutral attitude, which aligns closely with the ideal score of five. This group appears to possess a more balanced understanding, which is crucial for making informed decisions about SLT usage. Their education level likely equips them with a better grasp of the health implications, though their neutral stance may still indicate a degree of ambivalence toward the issue. Graduates show a mean STAARK score of 5.51, the highest among the groups, suggesting very good knowledge and a potentially lenient attitude toward SLT. This could indicate that while graduates are well-informed, their understanding might lead them to a more permissive view, which could be problematic in public health contexts. Professionals, with a mean STAARK score of 5.34, also demonstrate good knowledge but with a similar trend of a neutral to lenient attitude. This group’s educational background likely contributes to a solid understanding, but their scores suggest that more emphasis might be needed on fostering a preventative attitude toward SLT.

Effectiveness of the program

The study shows a significant positive impact of the SLT awareness program on the participants' knowledge and attitudes. The improvement in knowledge scores from the first to the second assessment indicates that the program successfully educated participants about the risks associated with SLT use. The strong correlation between knowledge and attitudes underscores the importance of educational components in awareness programs. The finding that improved knowledge significantly correlates with more negative attitudes toward SLT suggests that increasing factual understanding is key to changing perceptions. However, the slightly lower correlation in the second assessment points to the need for continual reinforcement of knowledge and attitude change. This highlights the importance of follow-up programs to maintain and deepen the impact of the initial intervention.

Risk assessment and recommendations

The 17 individuals filtered from the Assessment I population based on the STAARK score of above 6.1 were administered with a risk assessment survey on tobacco usage and were categorized individually. For four individuals at low risk (0-10 points), the primary focus was on prevention and maintaining a tobacco-free lifestyle. Educational interventions, awareness campaigns, and community engagement were the key strategies employed to reinforce healthy behavior and prevent the initiation of tobacco use. Six of those in the moderate risk category (11-20 points) were encouraged to reduce tobacco consumption with the goal of cessation. Motivational counseling, self-help resources, and behavioral interventions such as tracking usage and finding alternatives were recommended and advised. Four individuals in the high-risk category (21-30 points) were advised for a more urgent and stringent approach focused on cessation. Intensive counseling, personalized quit plans, and potentially nicotine replacement therapy or other pharmacological aids were crucially recommended. Comprehensive medical evaluations and peer support play a significant role in managing health concerns and sustaining motivation; thus, the Accredited Social Health Activists (ASHA) were instructed for further management of the identified 17 individuals based on the recommendations provided.

Challenges and limitations

The following are some of the challenges and limitations encountered in this study. The problem of response bias is the first limitation, as the STAARK scale is associated with the self-administered nature of the data collected through a survey. The participants may produce impressions that fit mainstream social norms with regard to knowledge or attitude toward SLT. Second, sample bias based on given responses of consecutive participants attending only one awareness program in Nagaland might restrict the validity of findings with other populations or other parts of the country with varying attitudes toward SLT. Additionally, while the study aims to assess the long-term impact of awareness programs, only two data collection points were used. This limits the ability to fully capture the sustained effects of the intervention over time. This restricts the examination of the extended impacts of the intervention over time and with varied levels of rigor. Fourth, a significant limitation of this study is its cross-sectional design, which captures data at a single point in time. This design prevents the study from establishing causal relationships between the SLT awareness program and changes in knowledge or attitudes. While the STAARK scale effectively measures knowledge and attitudes before and after the awareness program, the lack of longitudinal follow-up limits the ability to assess long-term changes in behavior or attitude. Additionally, the absence of a control group further weakens the study's ability to attribute observed changes in STAARK scores specifically to the intervention, as other external factors could influence the results. Future research could be designed to incorporate more diverse samples and to use more frequent assessments with a longitudinal design involving a control group in order to capture the stability of such changes.

Implications for future interventions

The scale can serve as an essential tool for healthcare providers, enabling them to assess the current level of awareness and knowledge of the risks of SLT across different populations. By identifying knowledge gaps, it helps tailor educational interventions to meet specific needs. Additionally, the scale evaluates attitudes and behaviors toward SLT, providing healthcare workers with insights into the motivations and barriers individuals face in quitting or reducing use. This understanding is crucial for developing targeted behavior change strategies. The data collected from the scale allows healthcare workers to create personalized cessation plans that address the unique needs and concerns of each individual, enhancing the effectiveness of intervention efforts. Furthermore, the aggregated data from the scale can be used to inform broader public health strategies, ensuring that resources are allocated efficiently. This information also aids in designing community-based interventions that directly tackle the most significant issues identified, making the scale a valuable resource for both individual patient care and public health planning.

ASHA, community health workers, and individuals with social healthcare responsibilities can benefit from using the STAARK scale during any awareness program in the context of tobacco control to advocate SLT cessation strategies in their local community. This can help them identify potential individuals vulnerable to tobacco usage who can be tailored for personalized counseling and cessation. The scale combined with risk assessment can be a vital tool for healthcare workers to identify individuals in need of rehabilitation therapy toward tobacco usage. This scale can be utilized in schools, which aids the teachers in identifying children and young adults in jeopardy due to tobacco usage and preventing them at the verge. This scale aids governmental and non-governmental organizations in improving the quality standards of their awareness programs and assessing their impact on the public on tobacco use.

The scale serves as a versatile tool for addressing SLT use across various populations. Among teenagers, it helps identify key factors driving the initiation of tobacco use, facilitating the creation of targeted prevention programs. For adults, especially those in rural areas or lower socioeconomic groups, the scale pinpoints barriers to quitting, enabling the development of support systems tailored to these challenges. In workplace settings, the scale assesses the effectiveness of health programs and identifies environmental factors contributing to continued tobacco use, guiding the implementation of more effective interventions. Additionally, the scale can be adapted to consider cultural and regional differences, allowing healthcare workers to tailor interventions that resonate with specific communities. It is also valuable in longitudinal studies, tracking changes in tobacco use over time within specific populations and providing data on the long-term impact of public health campaigns. Furthermore, the scale can be used as a training tool for healthcare workers, helping them understand the complexities of SLT addiction and improving their counseling skills, ultimately enhancing their ability to support patients in quitting.

## Conclusions

The STAARK scale could serve as an essential tool for evaluating awareness programs on SLT, which combines knowledge and attitude scores for a comprehensive assessment and identifies vulnerable populations who are at risk of addiction. It offers a standardized way to measure participants' understanding and attitudes toward SLT, facilitating comparison across different groups and times. By identifying those with lenient attitudes toward SLT, the scale helps to target high-risk individuals for more effective and personalized interventions. Its reliability and validation ensure consistency in informing public health strategies. Furthermore, by examining changes in STAARK scores pre- and post-program, researchers can assess the program's impact on participant knowledge and attitudes, aiding in the refinement of future interventions. The STAARK scale’s robust, multi-dimensional approach is crucial for effectively reducing SLT use through tailored, evidence-based public health efforts. In conclusion, the study underscores the critical role of the STAARK scale in awareness programs in combating SLT use by shaping their behavior and recommending of personalized interventions and enhanced caseation of SLT usage. This scale can serve as a powerful tool in the fight against SLT-related health issues by assessing the individual’s comprehension. However, the journey toward eradicating SLT use requires ongoing commitment, adaptability, and a focus on long-term outcomes to truly transform knowledge into lasting change.

## Appendices

Appendix 1: knowledge and attitude assessment

**Table 13:** Survey questionnaire: demographics, knowledge, and attitude assessment Attitude questions evaluated the participant's attitudes toward gutkha usage using a Likert scale with either four-point or five-point response options. The total attitude score, ranging from 10 to 60, was classified into five categories: 10-19 for a strong avoidance or highly negative attitude, 20-29 for a moderate avoidance or negative attitude, 30-39 for a neutral attitude, 40-49 for a moderately lenient attitude, and 50-60 for a strongly lenient or positive attitude for SLT usage. Higher scores demonstrated a more lenient attitude toward gutkha usage. These scores were further scaled for attitude and credited with one point for strong avoidance/highly negative attitude, two for moderately negative attitude, three for neutral attitude towards SLT usage, four for a lenient attitude, and five for a strong positive attitude towards SLT usage. Knowledge scores ranged from 0 to 10, categorized as follows: zero to three indicating low knowledge, four to six representing moderate knowledge, seven to eight reflecting good knowledge, and nine to 10 denoting excellent knowledge. These categories were then scaled for knowledge on a point basis as one for low knowledge, two for moderate knowledge, three for good knowledge, and four for excellent knowledge. cm: centimeters, Kg: kilograms

This study aims to assess the effectiveness of public health strategies in raising awareness about the risks associated with smokeless tobacco (SLT) use, particularly gutkha. We have developed a new tool, the Smokeless Tobacco Awareness, Attitude, and Response Knowledge (STAARK) Scale, to measure your knowledge and attitudes toward SLT before and after participating in the awareness program. You will be asked to complete a survey that includes questions about your demographic information, knowledge about SLT, and your attitudes toward its use. The survey is divided into three sections: demographic information: basic details like age, education level, etc.; knowledge assessment: multiple-choice questions about SLT and its effects; and attitude questionnaire: questions about your personal views on SLT use, rated on a scale. Your input is vital to helping us understand how effective our awareness programs are and where improvements can be made. By participating, you contribute to public health efforts aimed at reducing the harmful impact of SLT in our communities. I have read the information provided regarding the SLT awareness program evaluation study. I understand that the purpose of this research is to evaluate public health strategies by developing and validating the STAARK scale. The study will assess my knowledge and attitudes toward SLT usage before and after participating in the awareness program. I voluntarily agree to participate in this study. I understand that my participation involves completing surveys that include questions about my demographics, knowledge, and attitudes toward SLT. I acknowledge that my responses will be used to help identify gaps in awareness and design targeted interventions to reduce the use of SLT, particularly gutka.	
Question	Response options or data
Demographic questions	
1. What's your age?	(Enter age)
2. Education level	High school/higher secondary/graduate/professional
3. Height in cm	(Enter height)
4. Your weight in kg	(Enter weight)
Attitude questions	
1. Do you believe that gutkha usage is a serious health concern?	Strongly agree (1)/agree (2)/disagree (3)/strongly disagree (4)
2. Do you think people who use gutkha should seek help to quit?	Strongly agree (1)/agree (2)/disagree (3)/strongly disagree (4)
3. Is it acceptable to use gutkha in public places?	Strongly agree (1)/agree (2)/disagree (3)/strongly disagree (4)
4. How much do you think gutkha contributes to social problems?	Very significantly (1)/significantly (2)/moderately (3)/slightly (4)/not at all (5)
5. Do you believe that peer pressure is a significant factor in gutkha usage?	Strongly agree (1)/agree (2)/disagree (3)/strongly disagree (4)
6. What is your stance on using public funds for gutkha cessation programs?	Strongly support (1)/support (2)/neutral (3)/oppose (4)/strongly oppose (5)
7. How important is it to educate children about the dangers of gutkha?	Extremely important (1)/very important (2)/moderately important (3)/slightly important (4)/not important (5)
8. Do you believe that gutkha advertisements should be banned?	Strongly agree (1)/agree (2)/disagree (3)/strongly disagree (4)
9. Is gutkha usage viewed negatively in your community?	Strongly agree (1)/agree (2)/disagree (3)/strongly disagree (4)
10. Do you think quitting gutkha can improve a person's quality of life?	Strongly agree (1)/agree (2)/disagree (3)/strongly disagree (4)
Knowledge assessment	
1. What are the primary ingredients in gutkha?	a) Tobacco, areca nut, slaked lime, and flavoring agents / b) Tobacco, sugar, milk, and flavoring agents / c) Herbs, vitamins, minerals, and water / d) Fruits, nuts, honey, and spices
2. Which ingredient in gutkha is primarily responsible for its carcinogenic properties?	a) Areca nut / b) Slaked lime / c) Flavoring agents / d) Tobacco
3. What is a major health risk associated with prolonged gutkha usage?	a) Obesity / b) Oral cancer / c) Hypertension / d) Asthma
4. How does gutkha usage impact oral health?	a) Strengthens teeth and gums / b) Causes tooth decay, gum disease, and oral lesions / c) No significant impact / d) Prevents cavities
5. Which organ is most likely to be affected by harmful chemicals in gutkha?	a) Lungs / b) Liver / c) Heart / d) Mouth
6. What is an effective strategy to prevent gutkha usage among teenagers?	a) Offering free samples / b) Educating them about the risks / c) Advertising it as a stress reliever / d) Selling it at a discount
7. What role can schools play in preventing gutkha usage?	a) Distributing gutkha as rewards / b) Conducting awareness programs and providing educational resources / c) Ignoring the issue / d) Promoting it during sports events
8. Which of the following is a commonly recommended method to help someone quit gutkha?	a) Cold turkey (immediate cessation) / b) Gradual reduction / c) Nicotine replacement therapy / d) All of the above
9. What type of support is most beneficial for individuals trying to quit gutkha?	a) Peer support groups / b) Financial incentives / c) Access to gyms / d) Encouraging increased gutkha usage
10. What is a key component of a successful rehabilitation program for gutkha users?	a) Comprehensive education about the risks / b) Availability of gutkha at reduced prices / c) Isolation from friends and family / d) Ignoring withdrawal symptoms

Appendix 2: integrated risk assessment

**Table 14:** IRA questionnaires with points Risk assessment was performed using the IRA questionnaires with varying points system grading from 0 to 41. The IRA score classified participants into four risk categories: low (0-10), moderate (11-20), high (21-30), and very high (31-41). IRA: integrated risk assessment

Question	Response options	Points
1. How long have you used gutkha or other tobacco products?	Never	0
	Less than 1 year	2
	1-5 years	4
	5-10 years	6
	More than 10 years	8
2. How often do you use gutkha each day?	Never	0
	Less than once a day	1
	1-2 times per day	3
	3-5 times per day	5
	More than 5 times per day	7
3. How much gutkha do you use each time?	None	0
	A small amount (less than 1 pouch)	2
	A moderate amount (1-2 pouches)	4
	A large amount (more than 2 pouches)	6
4. What type of tobacco do you use?	None	0
	Less harmful (plain chewing tobacco)	2
	Moderate risk (gutkha with fewer ingredients)	4
	High-risk (gutkha with many harmful ingredients)	6
5. How is your mouth health?	Healthy	0
	Mild sores occurring rarely (e.g., occasional sores)	2
	Moderate sores occurring frequently (e.g., frequent sores)	4
	Severe with prominent mouth ulcers (e.g., mouth ulcers)	6
6. Does anyone in your family have habitual tobacco usage?	No	0
	Yes, primary circle relatives (mother, father, siblings)	4
	Yes, secondary circle relatives (relations other than primary circle)	2
7. Do you have other habitual health risks (e.g., alcohol use, poor diet, drug abuse)?	No	0
	Yes, one other risk	2
	Yes, more than one other risk	4
